# Nutritional programming of coenzyme Q: potential for prevention and intervention?

**DOI:** 10.1096/fj.14-259473

**Published:** 2014-12

**Authors:** Jane L. Tarry-Adkins, Denise S. Fernandez-Twinn, Jian-Hua Chen, Iain P. Hargreaves, Malgorzata S. Martin-Gronert, Josie M. McConnell, Susan E. Ozanne

**Affiliations:** * Metabolic Research Laboratories, Institute of Metabolic Science, University of Cambridge, Cambridge, UK; and; † Neurometabolic Unit, National Hospital, University College London, London, UK

**Keywords:** telomeres, cardiovascular disease, aging, vascular disease

## Abstract

Low birth weight and rapid postnatal growth increases risk of cardiovascular-disease (CVD); however, underlying mechanisms are poorly understood. Previously, we demonstrated that rats exposed to a low-protein diet *in utero* that underwent postnatal catch-up growth (recuperated) have a programmed deficit in cardiac coenzyme Q (CoQ) that was associated with accelerated cardiac aging. It is unknown whether this deficit occurs in all tissues, including those that are clinically accessible. We investigated whether aortic and white blood cell (WBC) CoQ is programmed by suboptimal early nutrition and whether postweaning dietary supplementation with CoQ could prevent programmed accelerated aging. Recuperated male rats had reduced aortic CoQ [22 d (35±8.4%; *P*<0.05); 12 m (53±8.8%; *P*<0.05)], accelerated aortic telomere shortening (*P*<0.01), increased DNA damage (79±13% increase in nei-endonucleaseVIII-like-1), increased oxidative stress (458±67% increase in NAPDH-oxidase-4; *P*<0.001), and decreased mitochondrial complex II-III activity (*P*<0.05). Postweaning dietary supplementation with CoQ prevented these detrimental programming effects. Recuperated WBCs also had reduced CoQ (74±5.8%; *P*<0.05). Notably, WBC CoQ levels correlated with aortic telomere-length (*P*<0.0001) suggesting its potential as a diagnostic marker of vascular aging. We conclude that early intervention with CoQ in at-risk individuals may be a cost-effective and safe way of reducing the global burden of CVDs.—Tarry-Adkins, J. L., Fernandez-Twinn, D. S., Chen, J.-H., Hargreaves, I. P., Martin-Gronert, M. S., McConnell, J. M., Ozanne, S. E. Nutritional programming of coenzyme Q: potential for prevention and intervention?

It has been known for several years that low birth weight is strongly associated with increased risk of cardiovascular disease (CVD) in later life (1, 2). Furthermore, risk of CVD and its associated metabolic dysfunction is exacerbated in low-birth-weight babies who experienced rapid postnatal growth (3–5). Animal models have provided valuable insights into potential underlying molecular mechanisms that link suboptimal maternal exposures to later outcomes of cardiovascular health. These include reductions in cardiomyocyte numbers at birth (6); structural changes, including alterations in aortic wall thickness (7); and increases in cardiac fibrosis (8) and increased cardiac oxidative stress (9).

Excessive reactive oxygen species (ROS) is known to damage cellular macromolecules such as proteins, lipids and DNA, if cellular antioxidant defenses are insufficient to maintain redox homeostasis. In particular, ROS accelerate telomere shortening in somatic cells (10, 11) by preferentially damaging the guanine-rich repeat sequences within telomeric DNA. Telomeres shorten after every somatic cell division, and in many species, including birds (12, 13), mice (13), and humans (13), telomere length has been correlated with longevity. Furthermore, telomere length plays a pivotal role in the onset, development, and prognosis of CVD (14). In humans, the aorta is a major site of telomere attrition (15), and aortic telomere length has been shown to be negatively correlated with age and atherosclerotic grade (15, 16). Using a well-established rat model of nutritionally induced low birth weight followed by accelerated postnatal growth (recuperated or catch-up growth model), we have previously demonstrated that these growth patterns are associated with reduced longevity compared with controls (17) and significant reductions in kidney (17), aortic (18), pancreatic islet (19), and cardiac (20) telomere length. The reductions in renal and cardiac telomere length were linked to a programmed deficit in renal (21) and cardiac coenzyme Q_9_ (CoQ_9_) levels (20) in later life.

The CoQ (or ubiquinone) molecule consists of a benzoquinone ring linked to an isoprenoid side chain, the length of which varies between species. In humans, the most common form is CoQ_10_, containing 10 isoprenoid units, whereas CoQ_9_ (containing 9 isoprenoid units) is the most common isoform in rodents (although rodents can convert dietary CoQ_10_ into CoQ_9_). CoQ acts as an electron carrier, shuttling electrons between complexes I and III and complexes II and III of the mitochondrial electron transport chain (ETC). In its reduced form (ubiquinol), it is a potent antioxidant, preventing initiation and propagation of lipid peroxidation (22). We have recently demonstrated that dietary supplementation with CoQ_10_ can ameliorate indexes of cardiac aging in rats exposed to catch-up growth by preventing accelerated cardiac telomere shortening, premature induction of p21 and p53 (mediators of cell senescence), and induction of apoptotic markers (20). Notably, CoQ_10_ supplementation exhibited no detrimental effects on control offspring (20).

Our previous studies have thus demonstrated that suboptimal nutrition in early life can lead to a programmed deficit in renal and cardiac CoQ_9_ in later life. However, it is unknown whether this deficit is tissue specific or is present in all tissues, most notably including those that are clinically accessible. Furthermore, it is unknown whether CoQ deficiency is a very early consequence of a suboptimal early environment and therefore likely to be a causative factor in mediating detrimental consequences in the offspring. Therefore, this study aimed to investigate the effects of poor maternal nutrition followed by rapid postnatal catch-up growth on CoQ_9_ levels and molecular markers of aging in aortic tissue at weaning; determine whether supplementation of a clinically relevant dose of dietary CoQ_10_ could restore any observed deficit in aortic CoQ_9_ and therefore correct molecular indexes of accelerated aging in later postnatal life; and establish whether levels of CoQ_9_ were also programmed in white blood cells (WBCs) and therefore identify its potential value as a diagnostic tool for assessing CVD susceptibility in later life and provide rationale for intervention in high risk individuals.

## MATERIALS AND METHODS

### Animal experimental groups

All procedures involving animals were conducted under the British Animals (Scientific Procedures) Act (1986). Pregnant Wistar rats were maintained on a 20% protein (control) diet or an isocaloric low-protein (LP; 8%) diet fed *ad libitum*, as described previously (23). Both diets were purchased from Arie Blok (Woerden, The Netherlands). The day of birth was recorded as d 1 of postnatal life. Pups born to LP-diet-fed dams were cross fostered to control-fed mothers on postnatal d 3 to create a recuperated litter. Each recuperated litter was culled to 4 male pups at random to maximize their plane of nutrition. The control group was the offspring of mothers fed the 20% protein diet and suckled by dams fed the 20% protein diet. Each control litter was culled to 8 pups. To prevent any stress to the animals when cross fostered, pups were transferred with some of their own bedding. Body weights were recorded at postnatal d 3, 7, 14, and 21 and at 12 mo. At 21 d, 2 males/litter were weaned onto standard laboratory chow (Special Diet Services, Witham, UK) and the other 2 were weaned onto the same diet supplemented with CoQ_10_ to give a dose of 1 mg/kg body weight/d. Animals were maintained on these diets until 12 mo of age. A further cohort of animals (control and recuperated offspring without CoQ_10_ supplementation) was weaned at 21 d of age, denied access to food overnight, and killed at 22 d of age. All animals were killed by CO_2_ asphyxiation. At postmortem, aortic tissue was removed, weighed, and snap-frozen in liquid nitrogen and then stored at −80°C until analysis. For all measurements, 1 pup/litter was used; thus, *n* represents number of litters throughout. Only male animals were used in this study.

### CoQ_10_ diet preparation

A dose of CoQ_10_ (1 mg/kg body weight/d; refs. 24–27) was used in this study. This was achieved by appropriate CoQ_10_ supplementation of laboratory chow, as we have described previously (20). Diet was prepared 2×/wk throughout the study.

### CoQ_9_ and CoQ_10_ measurement

Total tissue ubiquinone (CoQ_9_ and CoQ_10_) status was quantified in whole aortic tissue by reverse-phase HPLC with ultraviolet (UV) detection at 275 nm as described previously (20). CoQ_10_ was separated on an HPLC column (Techsphere ODS; 5 μm, 150×4.6 mm; Capital Analytical Ltd., Leeds, UK). The mobile phase consisted of ethanol:methanol:60% (v/v) perchloric acid; 700:300:1.2 (v/v) to which was added 7 g of sodium perchlorate (20). The flow rate was maintained at 0.7 ml/min, (20).

### WBC isolation

Samples of whole blood (10 ml) were obtained *via* cardiac puncture and added to tubes containing 1% 0.5 M EDTA (pH 8.0) and shaken. Samples were then divided equally into 4 tubes. Five volumes of red blood cell (RBC) lysis buffer [150 mM ammonium chloride, 10 mM potassium bicarbonate, and 0.1 mM EDTA (500 mM); pH 8.0] was added to each tube. Samples were incubated at room temperature for 5 min, vortexed, and then centrifuged at 4°C for 10 min at 10,000 *g*. Supernatant containing RBCs was removed and discarded to leave WBC pellets. The pellets were then cleaned by adding 2 vol of RBC buffer, mixed, and centrifuged as above. After removal of the supernatant, 1 ml of RBC buffer was added to the pellets and mixed. The WBCs were counted using a cell counter (Countess Automated Cell Counter; Invitrogen, Paisley, UK). WBC pellets were then centrifuged, and the supernatant was removed, snap-frozen, and stored at −80°C until analysis.

### Mitochondrial complex activities

All mitochondrial complex activities were measured at 30°C on the Uvikon XL spectrophotometer (Kontron Instruments, Ltd., Watford, UK). Before assay, all sample homogenates of whole aortic tissue were subjected to 3 freeze-thaw cycles to disrupt the mitochondrial membranes and allow substrates access to the active sites of the enzymes. Activities of complex I (NADH:ubiquinone reductase; EC 1.6.5.3), complex II–III (succinate:cytochrome *c* reductase; EC 1.3.5.1+EC 1.10.2.2), and complex IV as well as citrate synthase (CS; EC 1.1.1.27) activity were assayed as described previously (9). As CS is a mitochondrial marker enzyme, all complex activities were expressed as a ratio to CS to compensate for differences in mitochondrial enrichment in the cell samples.

### Telomere length analysis

High-molecular-weight DNA was extracted from whole aortic tissue using the Wizard Genomic DNA Isolation kit (Promega, Southampton, UK) according to the manufacturer's instructions. DNA quantity and purity were determined using a Nanodrop spectrophotometer (Nanodrop Technologies, Wilmington, DE, USA). DNA (1.2 μg) was digested by *Hinf*I and *Rsa*1 restriction enzymes, separated by pulsed field gel electrophoresis (PFGE). The restricted DNA samples were quenched with 5× SDS loading buffer and loaded onto agarose gels containing SYBR safe stain (Invitrogen). Gels were checked for nonspecific degradation of an undigested DNA control and complete digestion of the enzyme-restricted DNA by visualizing the stained gels under UV light using Gel Doc visualization software (Syngene, Cambridge, UK). The separated DNA was transferred onto nylon membranes by Southern blotting. Telomere length was measured using Telo TAGGG telomere length assays (Roche Diagnostics, Mannheim, Germany; ref. 9). Telomere signals were analyzed using Adobe Photoshop (Adobe Systems, Inc., San Jose, CA, USA) and MacBas software (Fujifilm UK, Bedford, UK). Telomere length was quantified as described previously (9).

### Markers of oxidative stress and antioxidant defense capacity

Western blotting analysis of whole aortic tissue was used to determine protein expression of nei endonuclease VIII-like 1 (NEIL-1), nicotinamide adenine dinucleotide diphosphate (NADPH) oxidase 4 (NOX-4), xanthine oxidase (XO), manganese superoxidase-dismutase (MnSOD), and catalase. Protein was extracted and assayed as described previously (9), and 20 μg protein was loaded onto 10, 12, or 15% polyacrylamide gels, dependent on the molecular weight of the protein to be measured. The samples were then electrophoresed and transferred to polyvinylidene fluoride membranes (9), using the following concentrations: NEIL-1, 1:500 (Novus Biologicals, Littleton, CO, USA); MnSOD, 1:10,000 (Upstate Biochemicals, Watford, UK); and catalase, 1:10,000 (Abcam, Cambridge, Cambridgeshire, UK) using anti-rabbit IgG secondary antibodies. XO (1:200; Santa Cruz Biotechnology, Heidelberg, Germany) was detected using anti-mouse IgGs. NOX-4 (1:200; Santa Cruz Biotechnology) was detected using anti-goat IgGs. Equal protein loading was confirmed by staining electrophoresed gels with Coomassie blue to visualize total protein.

### Statistical analysis

Where appropriate, data were analyzed either using a 3-way ANOVA with maternal diet, CoQ_10_ supplementation, and age as the independent variables. Otherwise, a 2-way ANOVA was used with maternal diet and age as the independent variables. Data are represented as means ± sem. A value of *P* < 0.05 was considered statistically significant. All statistical analyses were performed using Statistica 7 software (Statsoft, Inc., Milton Keynes, UK). In all cases, *n* refers to the number of litters.

## RESULTS

### Recuperated animals were born small and underwent rapid postnatal growth

**Figure 1.**
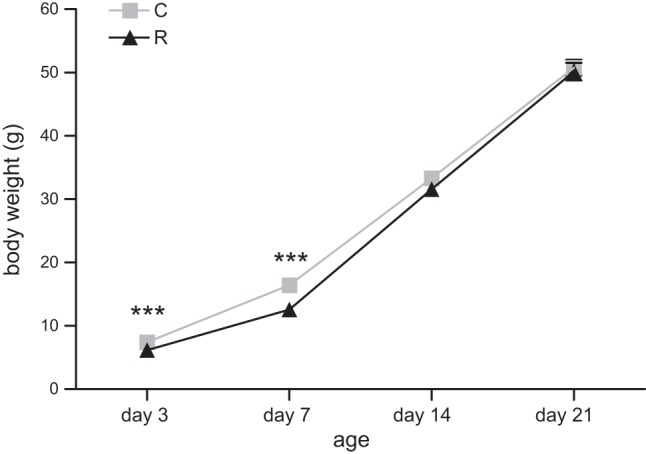
Effect of *in utero* protein restriction and accelerated postnatal growth on preweaning body weights. Results are expressed as means ± sem. ***P < 0.001 for recuperated (R) *vs*. control (C); *n*=10/group.

At postnatal d 3 and 7, recuperated pups were significantly (*P*<0.001) lighter compared with control offspring. However, by postnatal d 21, this group had undergone accelerated postnatal growth; therefore, the body weights were similar between groups (**Fig. 1**). At 12 mo of age, body weight remained similar between groups (**Table 1**).

**Table 1.** Group body weights

Group	Body weight (g)
Control	977 ± 26.7
Recuperated	937 ± 29.8
Control CoQ	1002 ± 38.0
Recuperated CoQ	954 ± 30.7

### Aortic and WBC CoQ_9_ levels were reduced in recuperated offspring

**Figure 2.**
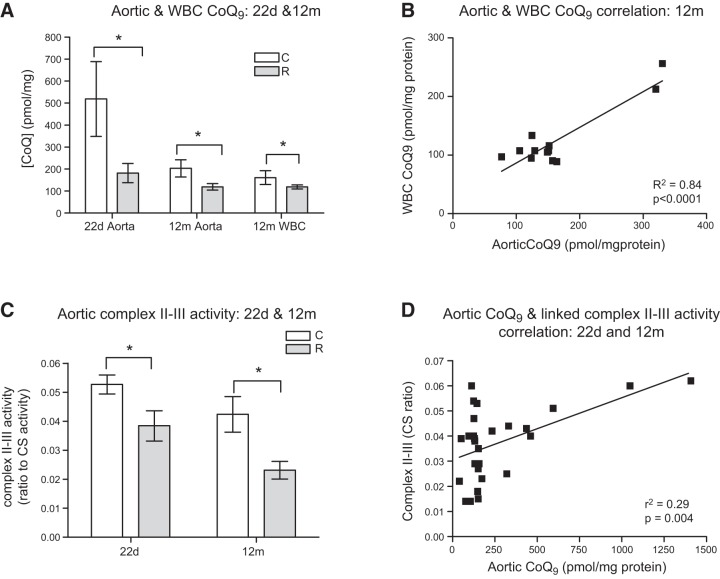
Effect of *in utero* protein restriction and accelerated postnatal growth on aortic and WBC CoQ_9_ levels in 22 d and 12 mo rats (*A*), correlation between aortic and WBC CoQ_9_ concentrations in 12 mo male rats (*B*), aortic linked complex II-III activity in 22 d and 12 mo male rats (*C*), and correlation between aortic CoQ_9_ levels and linked complex II-III activity in 12 mo male rats (*D*). Results are expressed as means ± sem. *P < 0.05; *n* = 6–8/group.

At 22 d of age, aortic CoQ_9_ concentration was significantly reduced (*P*<0.05) in the recuperated group compared with controls (**Fig. 2*A***). There was also an effect of aging such that CoQ_9_ levels in 12 mo aortas were significantly (*P*<0.05) lower than those observed at 22 d of age. At 12 mo of age, both aortic and WBC levels of CoQ_9_ were significantly reduced (*P*<0.05) in the recuperated group compared with control animals (Fig. 2*A*). CoQ_10_ supplementation had no effect on aortic CoQ_9_ levels in control animals (203±39 *vs.* 196±35 pmol/mg protein) nor in recuperated animals (119±15 *vs.* 158±30 pmol/mg protein). Likewise, CoQ_9_ levels in WBCs were also unaffected by CoQ_10_ supplementation (control: 161±32; control CoQ: 140±17; recuperated: 111±4; recuperated CoQ: 108±12 pmol/mg protein). Interestingly however, a strong positive correlation (*P*<0.0001; *r*^2^=0.84) was observed between aortic and WBC CoQ_9_ concentrations at 12 mo of age in control and recuperated offspring (Fig. 2*B*).

### Recuperated offspring had a deficit in linked complex II-III enzyme activity

Consistent with the CoQ_9_ deficit, a significant (*P*<0.05) reduction in linked complex II-III activity was observed in the recuperated group compared with controls at 22 d of age (Fig. 2*C*), whereas there was no difference in complex I (0.2±0.03 *vs.* 0.2±0.04 ratio to CS activity) or complex IV activity (0.01±0.001 *vs.* 0.01±0.001 ratio to CS activity). A deficit in linked complex II-III activity was still present at 12 mo of age (Fig. 2*C*) and CoQ_9_ levels were positively correlated with linked complex II-III activity; (*P*=0.004; *r*^2^=0.29) in control and recuperated offspring, (Fig. 2*D*). CoQ_10_ supplementation had no effect on complex II-III activity in the recuperated group (0.02±0.003 in unsupplemented *vs.* 0.02±0.003 in supplemented group; expressed as ratio to CS activity), however CoQ_10_ supplementation resulted in a significant (*P*<0.001) reduction in linked complex II-III activity in the control group (0.04±0.005 in unsupplemented *vs.* 0.02±0.003 in the supplemented group; ratio to CS activity).

### Indexes of oxidative stress were ameliorated by CoQ_10_ supplementation

**Figure 3.**
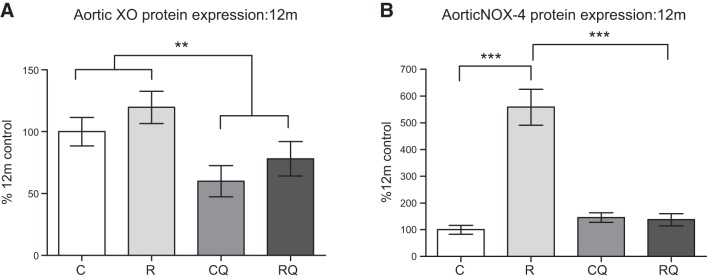
Effect of *in utero* protein restriction, accelerated postnatal growth, and CoQ_10_ supplementation on XO (*A*) and NOX-4 (*B*) protein expression in 12 mo male rats. C, control; CQ, control CoQ; R, recuperated; RQ, recuperated CoQ. Results are expressed as means ± sem. **P < 0.05, ***P < 0.001; *n* = 6/group.

At 12 mo of age, there was no significant effect of maternal diet on aortic XO protein expression; however, CoQ_10_ supplementation significantly (*P*<0.01) reduced XO levels (**Fig. 3*A***). NOX-4 protein levels were markedly (*P*<0.001) increased in the recuperated group compared with controls (Fig. 3*B*), an effect that was prevented by CoQ_10_ supplementation (*P*<0.001; Fig. 3*B*).

### CoQ_10_ supplementation prevents aortic telomere shortening in recuperated offspring

**Figure 4.**
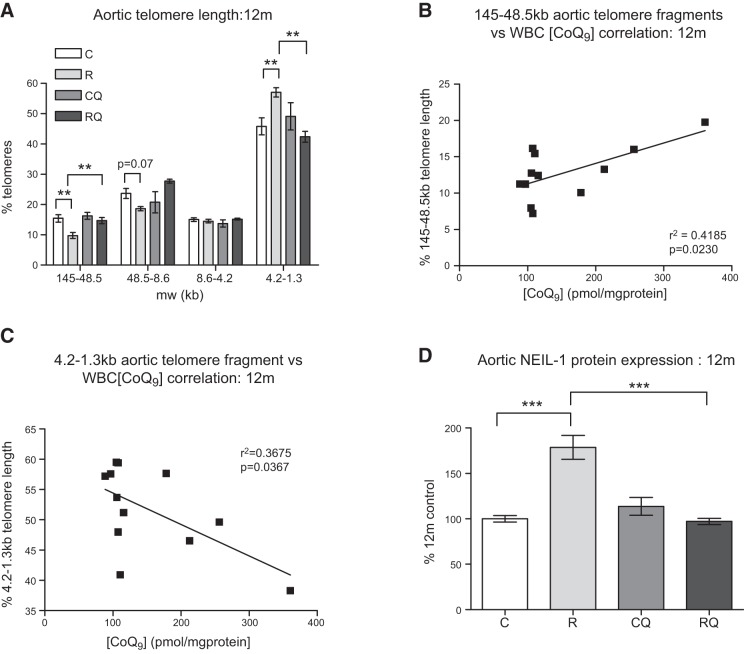
Effect of *in utero* protein restriction and accelerated postnatal growth on telomere length (*A*), correlation between aortic telomere length and WBC CoQ_9_ concentrations in 12 mo male rats (*B*, *C*), and NEIL-1 (*D*) protein expression in 12 mo male rat aortas. C, control; CQ, control CoQ; R, recuperated; RQ, recuperated CoQ. Results are expressed as means ± sem. **P < 0.01, ***P < 0.01; *n* = 6/group.

At 12 mo of age, recuperated animals had shorter aortic telomeres compared with controls, as reflected by significantly (*P*<0.01) fewer long (145–48.5 kb) and significantly (*P*<0.01) more short (4.2–1.3 kb) telomeres (**Fig. 4*A***). CoQ_10_ supplementation prevented the increased telomere shortening in the recuperated group (Fig. 4*A*). There was a positive correlation between WBC CoQ_9_ concentration and the proportion of the longest (145-48.5 kb) telomere fragments (*r*^2^=0.42; *P*<0.05; Fig. 4*B*) and a negative correlation with the proportion of the shortest (4.2-1.3 kb) telomere fragments (*r*^2^=0.37; *P*<0.05; Fig. 4*C*).

### CoQ_10_ supplementation abrogates up-regulation of NEIL-1, a base excision repair (BER) enzyme

At 22 d of age, NEIL-1 protein levels were significantly (*P*<0.05) increased in the recuperated group compared with controls (264±54 *vs.* 100±25%). Elevated NEIL-1 protein levels were maintained in the recuperated group at 12 mo of age (*P*<0.001; Fig. 4*D*). CoQ_10_ supplementation was able to prevent (*P*<0.001) this increase (Fig. 4*D*). Protein expression of Nthl endonuclease III-like-1 (NTHL-1) and 8 oxoguanine DNA glycosylase 1 (OGG-1) were undetectable at both ages.

### Antioxidant-defense capacity is altered in recuperated offspring and can be ameliorated by CoQ_10_ supplementation

**Figure 5.**
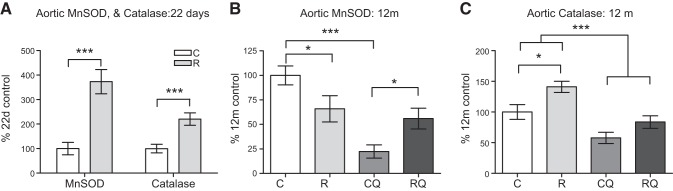
Effect of *in utero* protein restriction and accelerated postnatal growth on antioxidant protein expression in 22 d (*A*) and 12 mo (*B*, *C*) male rat aortas. C, control; CQ, control CoQ; R, recuperated; RQ, recuperated CoQ. Results are expressed as means ± sem. *P < 0.05, ***P < 0.001; *n* = 6/group.

At 22 d of age, MnSOD and catalase protein levels were significantly (*P*<0.001) increased in the recuperated group compared with controls (**Fig. 5*A***). At 12 mo of age, MnSOD levels were significantly reduced in the recuperated group; however, this decrease was not ameliorated by CoQ_10_ supplementation (Fig. 5*B*). Catalase levels (*P*<0.05) remained significantly elevated in the recuperated group compared with controls at 12 mo of age. However, CoQ_10_ supplementation significantly (*P*<0.001) decreased catalase protein expression (Fig. 5*C*).

## DISCUSSION

In this study, we demonstrated for the first time that an exposure to suboptimal nutrition during early life resulted in a deficit of aortic CoQ_9_ across the life course, which is associated with accelerated aortic telomere shortening. The normal life span for the rats used in this study is between 13 and 15 mo (28); therefore, the study of animals at 12 mo of age gives valuable insight into changes occurring toward the end of life. At 12 mo of age, aortic CoQ_9_ levels were ∼10 times lower than those previously reported in the heart (20) and lower in renal tissue (21). It is known that all tissues, with the exception of RBCs can synthesize CoQ; however, the levels of synthesis can vary greatly between tissues and this is thought to be largely dependent on how metabolically active and mitochondrially rich the tissue is. Therefore, lower aortic CoQ_9_ levels may reflect lower metabolic requirements of the aorta compared with the heart and may explain why CoQ_10_ supplementation was unable to significantly increase aortic CoQ_9_ status in either group.

Oxidative stress is a common feature observed in a number of models of developmental programming, including maternal hypoxia, maternal obesity, maternal protein restriction, and placental insufficiency (29). Oxidative stress can result from mitochondrial dysfunction and NOX and XO up-regulation (30) and has been strongly implicated in the pathogenesis of CVD (30). Poor maternal nutrition followed by rapid postnatal growth resulted in decreased linked complex II-III activity in aortic tissue, which was associated with increased levels of NOX-4, suggestive of a prooxidative phenotype in recuperated aortas. Increased oxidative stress is known to accelerate telomere shortening by preferentially damaging the guanosine-rich DNA sequences of telomeric DNA (31), and as a response to oxidatively damaged DNA, enzymes in the BER DNA damage pathway can be activated (32). Consistent with the observed increase in oxidative stress, we observed increased indexes of DNA damage in recuperated offspring, including increased levels of the BER enzyme NEIL-1 and accelerated aortic telomere shortening. Since the CoQ_9_ deficit is evident before telomere shortening (18), this supports a role for CoQ_9_ levels as an early programming mediator of telomere shortening, aging, and disease of the aorta.

Damage to DNA and other cellular macromolecules can occur if there is an imbalance between ROS generation and subsequent antioxidant defense capacity. At 22 d of age, expression of MnSOD [a mitochondrial specific antioxidant enzyme that is responsible for the conversion of the major cellular ROS. superoxide anion (O_2_^−^), into hydrogen peroxide (H_2_O_2_)] in recuperated offspring was significantly increased, however, by 12 mo of age, this effect was reversed, perhaps signifying that mitochondrial antioxidant defenses are compromised in older recuperated animals and aging is instrumental in the loss of MnSOD expression. A deficiency in MnSOD has been shown to increase mitochondrial oxidative stress and aggravate age-dependent vascular relaxation (33), and loss of this enzyme is a common phenotype of vasculature dysfunction (34, 35). Thus, the age-dependent loss of MnSOD may facilitate the observed oxidative stress in recuperated offspring. Levels of catalase (a nonmitochondrial antioxidant enzyme that catalyzes H_2_O_2_ into H_2_O and O_2_) remained elevated in recuperated offspring, which suggests that this compensatory response to oxidative stress is maintained. Indeed, it has previously been reported that this enzyme is up-regulated in sites of aortic coarctation, in the presence of oxidative stress (36). Taken together with the increased NOX-4 protein expression, reduced linked complex II-III activity, and CoQ_9_ deficit, this suggests a specific mitochondrial dysfunction in the aortas of recuperated offspring associated with an increase in oxidative stress.

CoQ_10_ supplementation was able to decrease oxidative stress by reducing NOX-4 and XO protein levels, restoring NEIL-1 protein to control levels, and critically, preventing accelerated telomere attrition. These findings support a direct role for oxidative stress in accelerated telomere attrition in recuperated offspring. Further support for this role comes from studies showing that MitoQ (a CoQ analog) can counteract fibroblast telomere shortening under mild oxidative stress conditions (37). CoQ_10_ supplementation did not, however, alter NOX-4, NEIL-1, or telomere length in the control group, which implies that CoQ_10_ supplementation is capable of ameliorating accelerated aortic aging only where a CoQ_9_ deficit exists. Notably, there was no adverse effect where CoQ_9_ levels were normal. While CoQ_10_ supplementation did not alter MnSOD levels in the recuperated group, catalase was reduced, which is likely due to its overall beneficial effects on the lowering of ROS levels (as evidenced by reduced XO and NOX-4).

Our studies have now shown that a CoQ deficit and compromised mitochondria occur in renal, heart, and aortic tissues of recuperated animals. Our initial simple in *in vitro* studies suggested that if CoQ levels were normalized then mitochondrial function would be fully restored (21). In the current *in vivo* work, where rats were supplemented with dietary CoQ, our findings suggest a complex and more indirect beneficial effect of high serum levels of CoQ. Premature aging is reversed by dietary CoQ, but tissue concentrations and mitochondrial activity are not corrected. Instead CoQ appears to induce the expression of additional beneficial antioxidant defenses.

Human meta-analyses have demonstrated that CoQ_10_ supplementation (doses ranging from 60 to 300 mg/d) can improve clinical outcome in patients with heart failure (38–40). Most notably, safety studies indicate that CoQ_10_ is well tolerated, has low toxicity, and does not induce serious adverse effects in humans. Risk assessments for CoQ_10_ based on various clinical trial data indicate that the observed safety level for CoQ_10_ in humans is between 900 and 1200 mg/d/person. Overall, these data from preclinical and clinical studies confirm that CoQ_10_ is safe for use as a dietary supplement (41, 42). In our study, we utilized a dose of 1 mg/kg body weight/d, a dose far lower than the reported maximum safe dose and one that has been previously tolerated without side effects (24–27).

Globally, CVD is responsible for more deaths than any other disease, claiming an estimated 17.3 million lives in 2008, a number that is predicted to grow to >23.3 million by 2030 (43). These statistics impress a critical need for the development of early biomarkers for CVD risk. For a biomarker to be feasible, it must be present in clinically accessible tissue. By measuring CoQ_9_ status in WBCs from control and recuperated rats, we demonstrated a significant CoQ_9_ deficiency in the WBCs of recuperated offspring. Notably, this strongly correlated to aortic CoQ_9_ levels. Furthermore, we also showed a highly significant relationship between CoQ_9_ levels and aortic telomere length in WBCs, suggesting that low WBC CoQ_9_ levels can predict short aortic telomeres and therefore susceptibility to aortic disease. These studies in rodents therefore have identified a biomarker in a clinically accessible tissue that has the potential to be used as a tool to identify individuals at risk of cardiovascular disease. A next step will be to establish whether these findings can be confirmed in humans and therefore make their prognostic potential a realistic possibility.

In summary, we have demonstrated, for the first time to our knowledge, that nutritionally induced low birth weight and catch-up growth leads to a programmed deficit in aortic CoQ_9_ that is coupled to increased DNA damage and telomere shortening. This accelerated aortic cellular aging can be prevented with dietary CoQ_10_ postweaning. The fact that CoQ_9_ levels were also programmed and detectable in blood raises the exciting possibility that WBC CoQ measurements could be prioritized as a marker of vascular aging and risk of CVD in later life. Early intervention with CoQ_10_ in identified at-risk individuals could therefore represent a safe and cost-effective treatment for cardiovascular disease.
